# Design and Evaluation of the Extended FBS Model Based Gaze-Control Power Wheelchair for Individuals Facing Manual Control Challenges

**DOI:** 10.3390/s23125571

**Published:** 2023-06-14

**Authors:** Xiaochen Zhang, Jiazhen Li, Lingling Jin, Jie Zhao, Qianbo Huang, Ziyang Song, Xinyu Liu, Ding-Bang Luh

**Affiliations:** 1 Department of Industrial Design, Guangdong University of Technology, Guangzhou 510090, China; xzhang@gdut.edu.cn (X.Z.);; 2 Guangdong International Center of Advanced Design, Guangdong University of Technology, Guangzhou 510090, China

**Keywords:** gaze control, power wheelchair, extended FBS model, assistive technology

## Abstract

This study addresses the challenges faced by individuals with upper limb disadvantages in operating power wheelchair joysticks by utilizing the extended Function–Behavior–Structure (FBS) model to identify design requirements for an alternative wheelchair control system. A gaze-controlled wheelchair system is proposed based on design requirements from the extended FBS model and prioritized using the MosCow method. This innovative system relies on the user’s natural gaze and comprises three levels: perception, decision making, and execution. The perception layer senses and acquires information from the environment, including user eye movements and driving context. The decision-making layer processes this information to determine the user’s intended direction, while the execution layer controls the wheelchair’s movement accordingly. The system’s effectiveness was validated through indoor field testing, with an average driving drift of less than 20 cm for participates. Additionally, the user experience scale revealed overall positive user experiences and perceptions of the system’s usability, ease of use, and satisfaction.

## 1. Introduction

The World Health Organization estimates that approximately 15% of the global population has some form of disability, with 2–4% experiencing severe functional difficulties [1]. Danemayer et al. [2] reported that the use of mobility devices (including prostheses, wheelchairs, intelligent power wheelchairs, etc.) ranged from 0.9 to 17.6% among patients with mobility challenges. As one of the most prevalent mobility aids, wheelchairs can significantly enhance the quality of life for people with disabilities and facilitate their participation in social activities.

However, individuals with hand or upper limb disadvantages may find it challenging to use conventional power wheelchairs. Cojocaru et al. [3] found that over half of powered wheelchair users experienced difficulties learning to maneuver a conventional powered wheelchair. To improve mobility for people with disabilities and promote dignified participation in social activities, researchers have explored various wheelchair interaction methods. A comprehensive guide by Marco et al. [4] explores the utilization of computer vision in healthcare to enhance the quality of life for individuals with disabilities. Moreover, a range of interaction techniques has been employed in power wheelchair human–computer interactions, such as voice control [5,6,7], Sip-and-Puff interfaces [8], Brain–Computer interfaces [9,10,11], Tongue Drive Systems [12], EMG-based interfaces [13,14], and eye-control interfaces [15,16,17]. These methods reduce the physical, perceptual, and cognitive skills required to operate a power wheelchair for a broader range of people with disabilities. However, some interaction methods face limitations in their commercialization potential due to factors such as the high environmental impact, control overloading, difficulty of use, and high cost.

With the widespread recognition and acceptance of user-centered design in recent years, humanized power wheelchair interaction systems have become the trend. Eye control is an intuitive and natural input method that many researchers have utilized in designing power wheelchairs. Power wheelchairs based on visual input are divided into blink-controlled and gaze-controlled. In the case of blink-controlled power wheelchairs, blinks are often used as signals to start and stop the system or to indicate the desired direction based on the number of blinks. Li et al. [18] designed an EOG-based switch with blinking as a condition for switching. If the user blinks in sync with the blinking of the switch button, the blink is judged to be intentional, and an on/off command is issued. Huang et al. [19] proposed a wheelchair robotic arm system that utilizes EEG and EOG signals. In their approach, blinking and eyebrow raising were employed as preselection and validation steps, respectively. The button can only trigger the corresponding command after it has been preselected and verified. Choudhari et al. [20] proposed a wheelchair system using one, two, and three voluntary blinks to control different commands: a single voluntary blink for forward and stop, two voluntary blinks for a left turn, and three voluntary blinks for a right turn. Blink-controlled power wheelchairs have a high degree of command accuracy, but frequent blinking may disrupt the user experience.

Eye-tracking wheelchair interaction systems using gaze control can be categorized into two types: gaze at the screen and gaze at the environment. Some researchers [17,21,22] have proposed systems that drive wheelchairs by gazing at a screen. In such systems, users can control the wheelchair by gazing at buttons or modules on a display, such as forward, left, right, back, and stop. Sunny et al. [23] developed a gaze control architecture for a wheelchair with a graphical user interface and a 6DoF robotic arm. The user can use the assisted robotic arm with the help of eye tracking and control the wheelchair to move by gazing at the wheelchair control interface on the graphical interface, which includes four buttons: forward, left, right, and backward. Although driving the wheelchair by looking at buttons on the display can increase the reliability of interpreting users’ intentions, there is a risk that users will need to shift their gaze from the environment to the display during use. Dahmani et al. [15] employed convolutional neural networks by inputting images of users’ eyes and processing them to determine the gaze direction to direct the wheelchair. However, this method uses two small cameras fixed to the frames of glasses to capture images of users’ eyes, with cameras facing users’ pupils, potentially causing discomfort. Ishizuka et al. [24] proposed a system based on gaze detection and environment recognition to enable movement in unknown environments by combining gaze information from eye tracking and obstacle information from LiDAR. However, the study’s experimental results primarily showed results related to turning, without elaborating on the overall movement effect.

Previous research indicates that eye-tracking technology is a natural and intuitive input method for individuals with disabilities to operate power wheelchairs, thereby improving their quality of life. However, gaps in research remain in terms of analyzing user requirements and the overall design process. To address these gaps, this study develops a gaze-controlled system for power wheelchairs using the extended FBS model and the MosCow method. The study analyzes the genuine needs of disabled users and describes the entire design process systematically. The system uses an eye-tracking device to detect users’ gaze positions and determine the wheelchair’s direction of motion. The system’s development and evaluation transpired in three phases, with aims and methods detailed in Table 1. In the first stage, literature research and user interviews identify potential user needs, and then functional requirements are defined through an extended FBS model. In the second stage, requirement prioritization is analyzed using the MosCow method, and the wheelchair system is designed to meet functional requirements based on M-level and S-level requirements. The final phase encompasses system evaluation.

In the subsequent sections, we will introduce and discuss each of these three stages.

## 2. Defining the Functional Requirements of the System

In this section, we conducted literature research and user interviews to gain a preliminary understanding of user needs for wheelchair interaction systems. To better address user needs, we employed the extended FBS model to analyze and transform the preliminary requirements, resulting in more scientific and objective user requirements. Throughout this process, we took into account the user’s basic situation, daily challenges, and expectations for future wheelchairs.

### 2.1. Method

#### 2.1.1. Literature Research

Yuan et al. [25] employed the Analytic Hierarchy Process (AHP) and Kano Model to categorize wheelchairs into three levels based on their features and characteristics. The three levels identified were low-level wheelchairs, which offer basic dimensions and affordability; mid-level wheelchairs, which emphasize comfort and cost effectiveness; and high-level wheelchairs, which provide comfort, optimal functionality, and innovation. Meanwhile, Rice et al. [26] examined falls among wheelchair users, many of whom require assistance to recover and may remain on the floor for 10 minutes or longer. Moreover, the researchers [27] conducted semi-structured interviews with a cohort of 20 wheelchair users, revealing that 70% of the participants expressed fear of falling, while 80% of them acknowledged requiring assistance for recovery. Pellichero et al. [28] also investigated wheelchair use safety. Frank et al. [29] conducted a study on the pain and discomfort experienced by power wheelchair users who believed their pain was related to the wheelchair. Additionally, Viswanathan et al. [30] conducted surveys and found that wheelchair users want the ability to choose different levels of smart wheelchair control based on their physical condition and scenarios while expressing concerns about safety. In a cross-sectional study based on personal interviews, Sarour et al. [31] identified safety, comfort, and weight as the paramount concerns among wheelchair users. Table 2 provides a comprehensive summary of these requirements. Users prioritize several key factors that greatly influence their wheelchair experience. Ensuring safety, optimizing comfort, maintaining cost effectiveness, and facilitating easy operation are the primary considerations that underscore their essential needs. By addressing these aspects, wheelchair designs can better meet the expectations and requirements of users, leading to enhanced satisfaction and overall usability.

#### 2.1.2. User Interviews

We interviewed two long-term users of power wheelchairs via online and telephone platforms. One participant had paraplegia while the other had progressive muscular dystrophy. Both individuals had more than 4 years of experience using wheelchairs and had used three different models. During the interviews, we inquired about their personal background, wheelchair usage experience, and future expectations for wheelchairs. Participants reported that wheelchairs are necessary for daily activities and provide significant assistance in daily life. Their primary concerns included collisions, rollovers, and obstacles encountered due to poor control and complex driving situations. They expressed a desire for future wheelchairs to be safer, more easily controllable, and more cost-effective. With respect to the gaze-controlled wheelchair, they expected it to be safe, accurate, easy to control, comfortable, visually appealing, and affordable.

#### 2.1.3. The Extended FBS Model

**Figure 1:**
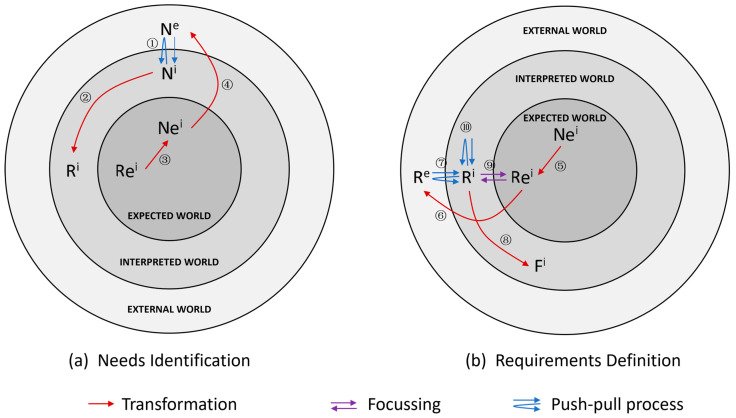
The Extended FBS model.

To circumvent the limitations of a traditional user requirement analysis through research and interviews, this study employed the Extended Function–Behavior–Structure (FBS) model for analyzing potential user requirements during the early stages of system design, providing a reference for subsequent design processes. Gero et al. [32,33] initially proposed the FBS framework, which describes design activities by connecting the external world, interpreted world, and expected world through three variables: Function (F), Behavior (B), and Structure (S) variables. The design activity’s outcome involves effecting change in the external world by focusing on the goals achieved in the expected world. Cascini et al. [31] further developed and refined the model by integrating two new variables, Needs (N) and Requirements (R), within the FBS framework’s three worlds, ultimately proposing the Extended FBS model [34], as illustrated in Figure 1.

In this context, ‘N^e^’ denotes ‘Needs in the External World’, while ‘N^i^’ signifies ‘Needs in the Interpreted World’ and ‘Ne^i^’ represents ‘Needs in the Expected World’. Similarly, ‘R^e^’ stands for ‘Requirements in the External World’, ‘R^i^’ refers to ‘Requirements in the Interpreted World’, and ‘Re^i^’ corresponds to ‘Requirements in the Expected World’. Lastly, ‘F^i^’ is indicative of ‘Functions in the Interpreted World’.

In the Extended FBS model, needs represent an expression of a perceived undesirable or ideal situation, which can be extracted by observing user behavior or perceived or assumed by the designer. Requirements refer to measurable attributes associated with one or more needs. The Extended FBS model describes the processes of need identification and requirement definition, consisting of the following steps:

- ① Investigating user needs N^e^ in the external world and generating interpretations of needs N^i^.
- ② transforming N^i^ into a preliminary requirement R^i^.
- ③ Translating the initially expected requirements Re^i^ into Ne^i^, ensuring that unprovided user needs are considered.
- ④ Transforming Ne^i^ into N^e^ and then verifying the expected requirements with the user and, if negative feedback is received, reinterpreting N^e^ through steps ① and ②.
- ⑤ Transforming Ne^i^ into Re^i^ variables.
- ⑥ Expanding Re^i^ to more or the equal number of R^e^.
- ⑦ Deriving and interpreting R^e^ into R^i^ (with the help of design experience).
- ⑧ Transforming part of R^i^ into F^i^.
- ⑨ Further focus on R^i^ as Re^i^ to obtain the initial design requirements.
- ⑩ Refining R^i^ through design experience and further derivation of new design requirements.

### 2.2. Results

**Figure 2:**
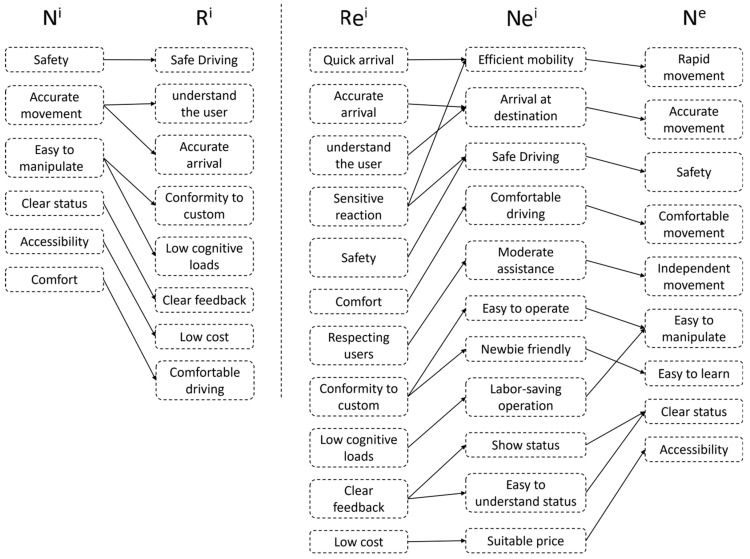
The results of the need identification.

To avoid the drawbacks of a traditional user needs analysis relying solely on research interviews, this study employed the Extended FBS model to identify the results of literature research and user interviews. First, the external user needs N^i^ obtained from the research are transformed into R^i^, which allows for the aggregation of needs by category. Second, Re^i^ is transformed into Ne^i^ by fully considering the necessary requirements not mentioned by the user, and then further verifying with the user to confirm the expected requirements. The results of the need identification based on the Extended FBS model are illustrated in Figure 2.

**Figure 3:**
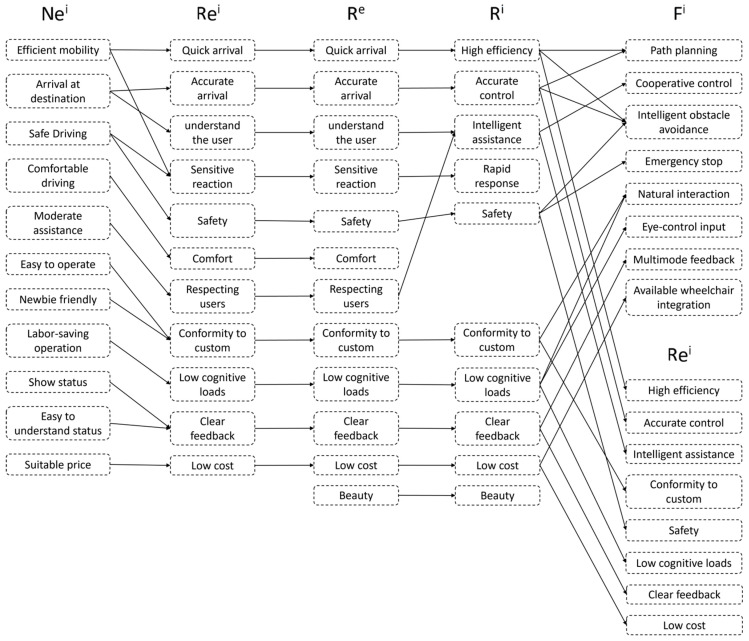
The results of the requirement definition.

Upon obtaining Ne^i^, the design requirements are defined, as illustrated in Figure 3. First, Re^i^ is transformed and expanded into R^e^; second, R^e^ is derived into R^i^ with the assistance of design experience; finally, a portion of R^i^ is converted into F^i^, and R^i^ is further refined into Re^i^ to obtain the initial design requirements.

According to Figure 3, users expected wheelchairs to include needs such as high efficiency, accurate control, intelligent assistance, safety, custom conformity, a low load, clear feedback, and a low cost. These expectations include functions such as path planning, collaborative control, intelligent obstacle avoidance, an emergency stop, natural interaction, eye-control input, multimodal feedback, and integration with existing wheelchairs.

### 2.3. Summary of This Section

This section aims to identify the needs and challenges of wheelchair users through literature research and user interviews. To ensure the objectivity and accuracy of the user requirements, we applied the extended FBS model to analyze and transform the preliminary requirements. Based on this, we established that the ideal wheelchair should possess the following needs: high efficiency, accurate control, intelligent assistance, safety, custom conformity, a low weight, clear feedback, and a low cost. Additionally, users expect the wheelchair to have several functions, including path planning, collaborative control, intelligent obstacle avoidance, an emergency stop, natural interaction, eye-controlled input, multimodal feedback, and integration with existing wheelchairs.

## 3. Realizing the Functional Requirements of the System

In this section, we detail the design and development of a prototype wheelchair system predicated on previously established requirements. Initially, we prioritized the F^i^ and Re^i^ identified earlier using the MosCow method. This categorization facilitated the delineation of “Must Have” and “Should Have” requirements, which subsequently guided the conceptualization and prototyping of the requisite wheelchair functionality.

### 3.1. Method

Upon obtaining the initial design requirements, it was important to prioritize them to facilitate subsequent design and prototyping. In order to prioritize the requirements effectively, we employed the MosCow method, a qualitative technique widely used in the industry. The MosCow method categorizes requirements into four priority levels: Must Have, Should Have, Could Have, and Won’t Have [35]. Must Have requirements are deemed crucial for the success of the project, and they must be implemented in the final product. Should Have requirements are important but not as critical as Must Have requirements, and they should be implemented if the time and budget allow. Could Have requirements are desirable but not essential, and they can be implemented if the time and budget permit. Won’t Have requirements are not included in the current project scope but may be considered in future iterations. Table 3 outlines the specific details of the prioritization process and the requirements categorized under each priority level. By using the MosCow method, we were able to prioritize the design requirements effectively and ensure that the final product met the essential needs of our users.

### 3.2. Results

#### 3.2.1. Priority of Requirements

To prioritize these requirements, we adopted the MosCow method, taking into account various crucial factors. These include the value, the feasibility of implementation, the impact on user satisfaction, and the cost effectiveness in terms of resources, time, and costs.

1. Value: Assess the importance of each requirement to the user to determine its level of importance in relation to the overall solution.
2. Feasibility: The feasibility of implementing a requirement is assessed by considering factors such as implementation difficulty, technical feasibility, and resource constraints.
3. User satisfaction: The impact of a requirement on the user experience is taken into account, with a focus on assessing its contribution to enhancing user satisfaction.
4. Cost effectiveness: Resources, time, and costs needed to fulfill the requirements are evaluated, along with careful consideration of their economic benefits in the context of the overall solution.
5. Timeliness: The urgency and time required to fulfill a requirement are carefully considered to determine its appropriate placement within the solution development cycle.

**Figure 4:**
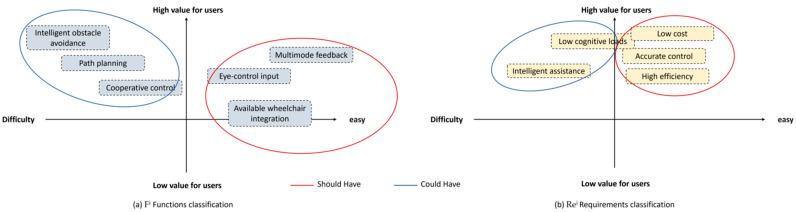
The four quadrants of value–difficulty rule.

Finally, we have categorized these requirements into two levels: M-level requirements and S&M-level requirements, as illustrated in Table 4. S-level and C-level requirements were then determined with the assistance of the value–difficulty rule’s four quadrants, as shown in Figure 4. The final MosCow requirement prioritization was obtained, as displayed in Table 5. The Must Have requirements of the system include conformity to custom, safety, and clear feedback, which are essential in ensuring the safety and satisfaction of the users. Additionally, the Must Have functions include natural interaction and an emergency stop, which allow for intuitive and safe control of the wheelchair in various situations. On the other hand, the Should Have requirements include high efficiency, accurate control, and a low cost, which are necessary to ensure that the system is practical and accessible to a wide range of users. Correspondingly, the Should Have functions include available wheelchair integration, multimode feedback, and eye-control input. By meeting these requirements and functions, the developed system can provide a reliable and efficient solution for individuals with mobility limitations, improving their quality of life and independence.

According to the MosCow method, the M-Level requirements are conformity to custom, safety, and clear feedback. M-Level functions are natural interaction and an emergency stop. Natural interaction can use eye-control input to control the wheelchair, which is one of the functions of the S-level. Additionally, eye-control input through staring at the environment is not only consistent with the user’s driving habits, but also enhances user safety by avoiding distraction from computer interfaces. The emergency stop function allows the user to stop the wheelchair autonomously under any circumstances to avoid accidental collisions. Clear feedback can be achieved through multimodal feedback (an S-level function), and the status of the wheelchair can be fed back to the user in real time with the help of vibration and the voice. In addition, the requirements of the S-level also include high efficiency, accurate control, and a low cost, which means that the structure of the system must be simple and can be integrated into the available wheelchair (an S-level function) to reduce unnecessary costs and expenses. At the same time, the information transmission of each device should be efficient. Natural interaction, feedback, and efficiency can be evaluated utilizing the System Usability Scale (SUS). Meanwhile, factors such as efficiency, accurate control, and safety can be gauged through simulated driving experiments, considering metrics such as the driving time, average drift, and instances of a collision or other accidental situations. Additionally, user satisfaction can be measured using satisfaction scales, facilitating an assessment of conformity to user customs.

#### 3.2.2. Sensors

**Figure 5:**
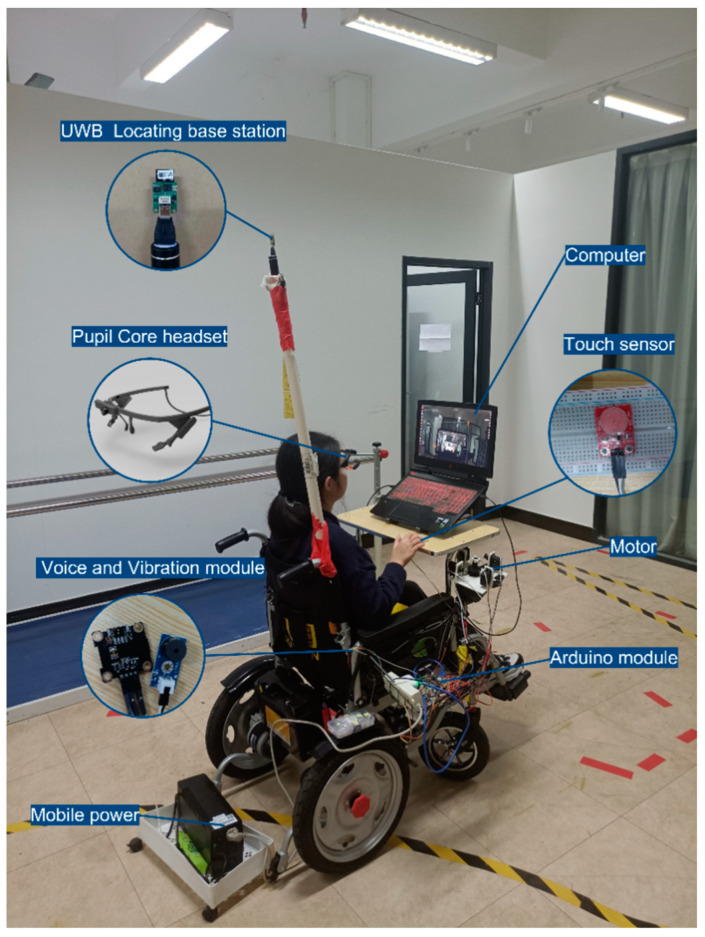
Proposed wheelchair prototype.

**Figure 6:**
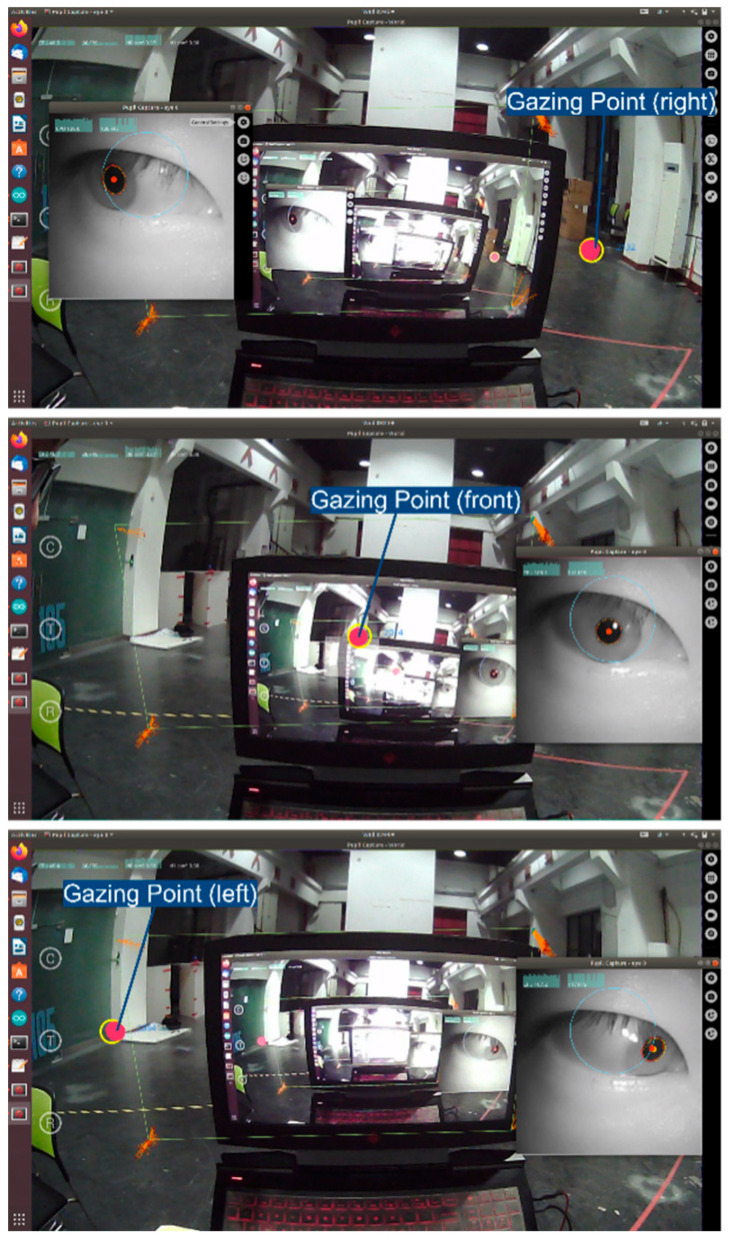
Eye tracker to obtain the gazing point.

The proposed gaze-controlled wheelchair system is based on a commercially available power wheelchair modified with touch sensors, control modules, audio and vibration modules, and an eye-tracking module, enabling precise control through gaze interaction. Figure 5 displays the sensors used. The eye tracker (Pupil Core headset) features a world camera and an eye camera. The Pupil Core headset serves as a user interface for the proposed interactive system, detecting the user’s gaze point in the environment to control the wheelchair’s direction of travel, as illustrated in Figure 6. The voice and vibration module utilizes hardware such as vibrating pads and a small buzzer to provide multimodal interaction for feedback on various system states, including the system wake-up/arrival/emergency stop, etc.

The control module comprises an Arduino module and four MG 995 motors, which control the wheelchair’s movement based on the gaze information obtained from the eye-tracking device. For instance, when the user gazes forward, the control module drives the wheelchair forward. When the user gazes left (right), the control module steers the wheelchair left (right).

The touch sensor can be mounted anywhere on the gaze-controlled wheelchair. The user can adjust the pressure sensor’s position according to their residual capacity to apply pressure for waking up the wheelchair and activating its emergency stop function by relieving the applied pressure. Further sensor details are shown in Table 6.

#### 3.2.3. Gaze-Controlled Wheelchair System Process

**Figure 7:**
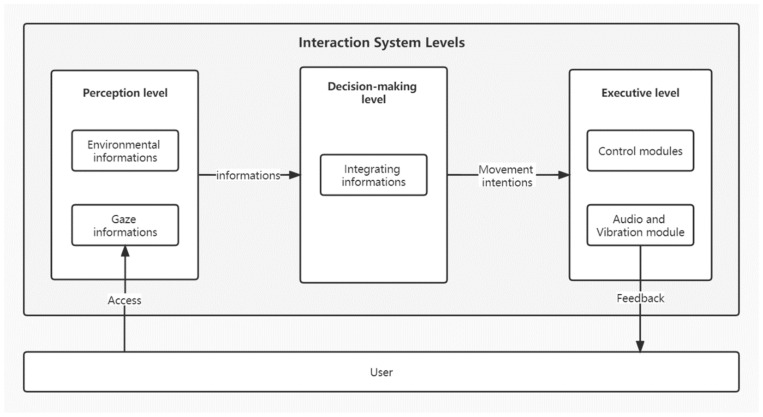
Interaction system level diagram.

The proposed gaze-controlled wheelchair system, based on the natural gaze of the environment, consists of three layers: perception, decision making, and execution. The perception layer is primarily responsible for sensing and acquiring surrounding information, including the user’s eye movements and driving environment information. The decision layer processes the information and data from the perception layer, integrating and analyzing the collected information to determine the end user’s intended movement (go forward, turn left, or turn right). The execution layer controls the wheelchair’s direction of movement, as determined by the decision layer, through the control module. Simultaneously, it provides feedback to the user about the wheelchair’s driving status through the audio and vibration module, as illustrated in Figure 7.

**Figure 8:**
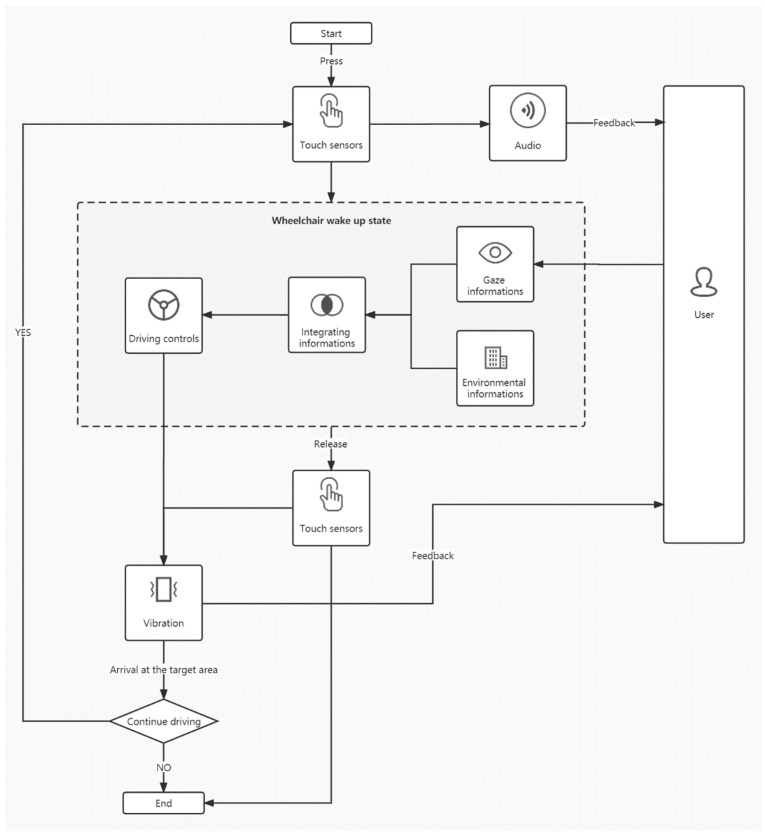
Flowchart of the interactive system.

The interaction system’s functional design is divided into two main modules: wake-up/emergency stop and driving. Prioritizing safety as the most critical M-level requirement, the wake-up/emergency stop control program’s logic supersedes that of the driving module. Only when the user applies pressure to the touch sensors can the gaze-controlled wheelchair be awakened and transition into the driving phase; the wheelchair receives the wake-up command while the user is informed of the current state through audio feedback. In any driving state, the user can release the pressure applied to the touch sensor to quickly disengage the wheelchair from the driving state and initiate an emergency stop for the user’s safety. Simultaneously, vibration feedback informs the user that the wheelchair has transitioned from the driving state to the stop state, as shown in Figure 8. To ensure clear and multimodal feedback, employing various feedback methods is crucial to alert the user when the wheelchair starts or stops, helping to prevent user confusion and satisfying the clear feedback requirement.

The driving module is the main functional module. The system covers most of the requirements in MosCow, such as controlling the wheelchair in a natural interactive way that conforms to the user’s habits, and efficiently captures the user’s driving intention information to drive the power wheelchairs to the target area. Specifically, the system detected the user’s eye movement information and determined the position of the user’s gaze point in the environment using an eye-tracking device. After obtaining the user’s intention to move, the control module drove the gaze-controlled wheelchair toward the target area. When the wheelchair reached the target area, it stopped moving and emitted vibrating feedback to indicate that the user had reached it. The user could then perform the next gaze, causing the wheelchair to move again. The user can make the wheelchair move several times over short distances by gazing at them, eventually achieving long-distance movement in the wheelchair.

#### 3.2.4. Implementation of the Gaze-Controlled Wheelchair System

In our study, we employed the widely used MATLAB software as a valuable tool for collecting and analyzing the gaze information obtained from the eye tracker. This powerful software allowed us to accurately capture and process the intricate data related to the participant’s eye movements.

Following the collection of gaze information, we established a seamless connection between the eye tracker and the Arduino board. The Arduino board effectively evaluated the received data and intelligently determined the appropriate actions based on the user’s gaze, including making decisions to turn left, turn right, or move forward.

To facilitate the translation of these decisions into physical movements, the Arduino board expertly regulated the movement of the wheelchair joystick by effectively controlling the motors. This enabled smooth and precise moving of the wheelchair in the intended direction. As the user approached the target position, they could effortlessly release the touch sensor, indicating their intention to halt the movement. In response, the Arduino board promptly commands the servo to bring the wheelchair to a stop, successfully concluding the movement.

Through this well-coordinated integration of software and hardware components, our proposed system effectively translated the user’s gaze information into accurate and responsive control of the wheelchair. This enhanced the overall usability and control of the wheelchair, enabling users to navigate their environment with ease and precision.

## 4. Evaluation and Results

This section focuses on testing and evaluating the prototype of the gaze-controlled wheelchair interaction system. To assess the effectiveness, usability, ease of use, and usefulness of the prototype, we conducted both an indoor simulated driving test and a System Usability Scale (SUS) evaluation. These tests aimed to evaluate the system’s performance and its ability to meet users’ needs, providing valuable feedback for future development and improvement.

### 4.1. Method

#### 4.1.1. Participants

In the third phase of the study, 14 participants (8 females and 6 males) were included. All participants had a normal or corrected-to-normal visual acuity greater than 4.5. Given that the system is currently in an early research phase, all participants enlisted for this simulation trial were individuals without disadvantages. This precaution was taken to preclude potential harm to users with disadvantages, considering the nascent state of the product.

#### 4.1.2. Experimental Site

**Figure 9:**
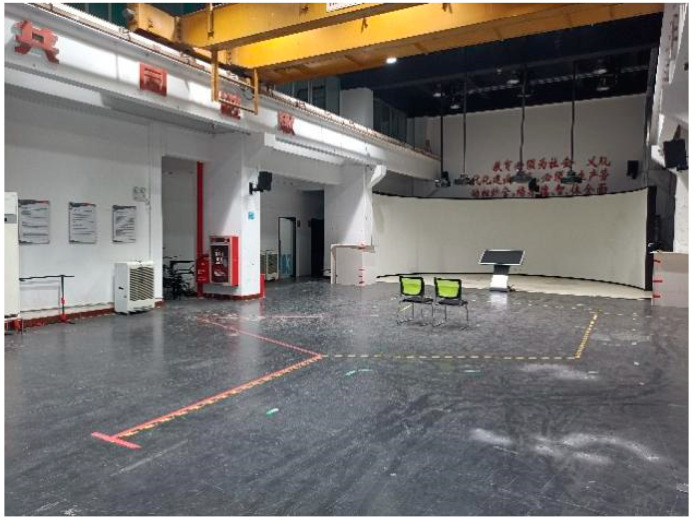
The real shot of the experimental site.

**Figure 10:**
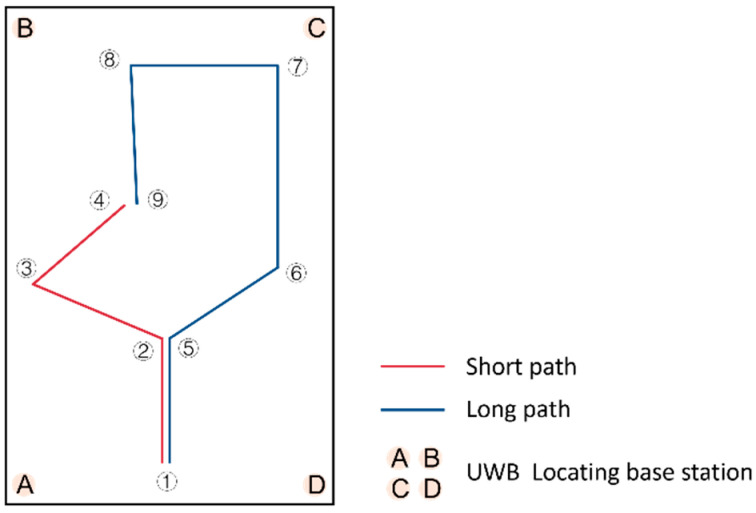
Schematic diagram of the site.

We consulted the publication by MacPhee et al. [36] for guidance on the arrangement of the experimental site. We selected an experimental site measuring 800 mm × 1215 mm. Based on daily driving needs, short and long experimental paths were planned and marked on the ground with red and yellow–black tape, including sharp, obtuse, and right-angle turns. While these two paths may not have covered all scenarios of wheelchair users’ daily travel activities, they represented common paths. The experimental site is shown in Figure 9, and the experimental site plan and planned paths are shown in Figure 10. The start and end points, along with the turning points, in the planned path are sequentially labeled with numbers to facilitate further discussion and analysis.

#### 4.1.3. Positioning Method

The subject’s travel path was recorded using the two-dimensional positioning mode of the Ultra-Wideband (UWB) indoor positioning system. An indoor positioning module was placed at each of the four corners of the experimental site and on the wheelchair, as shown in Figure 10. The positioning module at point A was marked as the primary base station, while B, C, and D were marked as secondary base stations. The positioning module on the wheelchair was marked as a tag. During the experiment, all base stations remained stationary. The tag moved with the wheelchair and communicated with the other four base stations, transmitting location information in real time to the primary base station, which was connected to a computer to output the tag’s location information.

#### 4.1.4. Evaluation Indicators

1. Time

The time factor, serving as a critical evaluation indicator, corresponds to the duration required for a user to perform a particular task using the system. This quantitative metric provides an objective assessment of both system efficiency and user proficiency.

2. Drift

Another critical performance measure is drift, which measures the system’s precision. It is defined as the average deviation from the target or expected result, thus providing insights into the accuracy of the system’s performance.

3. System usability scale

SUS is a tool for assessing the usability of a product or system. It was developed in 1996 by John Brooke [37]. The scale consists of ten declarative sentences, and users are asked to rate their agreement with each sentence after using the product or system. The odd-numbered items on the scale have positive statements, while the even-numbered items have negative statements. SUS is highly versatile and can be used to measure a wide range of user interfaces. In the field of power wheelchair research, many researchers have used SUS to score proposed systems.

Callejas-Cuervo et al. [38] designed a prototype wheelchair with head movement control and tested it using SUS. The final mean SUS score for the 10 subjects was 78. Guedira et al. [39] proposed a haptic interface for manipulating a wheelchair and gathered three wheelchair users to test it and score it on SUS. Panchea et al. [40] conducted a qualitative and quantitative study of an intelligent power wheelchair based on SUS. The results showed that the proposed graphical interface-based touch-controlled wheelchair SUS showed an average of 68.

4. Satisfaction questionnaire

The satisfaction questionnaire is an instrumental tool to quantify user satisfaction. It helps in understanding how well the system meets user expectations and provides a subjective measure of the system’s performance from the user’s perspective.

#### 4.1.5. Procedure

The specific procedure of the experiment was as follows:

Experiment description and operation instruction. The staff explained the experiment’s purpose, task, procedure, and precautions, helped the subject put on the eye tracker, calibrated it, and instructed the subject on the essential operation of the wheelchair and the feedback method.

Path driving. The subjects were asked to complete two different driving tasks of varying difficulty. From the initial point ①, the subject drove the prototype wheelchair along a short path to the endpoint ④. Then, they followed a long path from the initial point ① to the endpoint ⑨. Each subject was instructed to operate the eye-tracking wheelchair along the short path once, and subsequently complete two additional trials along the long path. At the same time, the staff observed and recorded the driving behavior and duration for each subject and ensured their safety.

Evaluation and reporting. At the end of the experiment, each participant was invited to fill in a user experience evaluation form and report on their experience of driving the gaze-controlled wheelchair.

### 4.2. Results

#### 4.2.1. Driving Function Evaluation

In the gaze-controlled wheelchair system driving evaluation experiment, 14 subjects successfully reached the endpoint in 42 path-driving tests. The observed and recorded driving conditions of the subjects are shown in Table 7. To minimize the influence of accidental conditions on the experimental results, the average driving time was calculated after removing the maximum and minimum values for the two paths, respectively. As displayed in Table 7 and Table 8, all subjects were generally able to drive normally during the driving sessions. The mean time for short-distance driving was 1.13 min, and the mean time for long-distance driving was 1.79 min.

The UWB positioner located the wheelchair at a 10 Hz frequency and transmitted its position coordinates to the computer in real time. After the experiment, the obtained coordinates of the wheelchair position were compared with the coordinates of the intended route, and the drifts between the two were the output. The average drifts for the 14 subjects for the 14 short and 20 long paths are shown in Table 9 and Table 10. From Table 9, the minimum drift for the short path was 9.37 cm, the maximum drift was 34.26 cm, and the average drift for the 10 tests was 19.49 cm. From Table 10, the minimum drift of the long path was 7.98 cm, and the maximum drift was 33.99 cm, with an average drift of 15.72 cm over 20 tests.

The average drift for the short path was greater than for the long path. According to the post-test reports, the larger drift in the short path trajectory was due to the initial unfamiliarity with the prototype gaze-controlled wheelchair interaction. During long path driving, the user could operate the gaze-controlled wheelchair more consistently and smoothly because of their experience with short path driving and increased familiarity with the system’s operation, feedback, and delay times. The average drift for the long path was also minimal.

**Figure 11:**
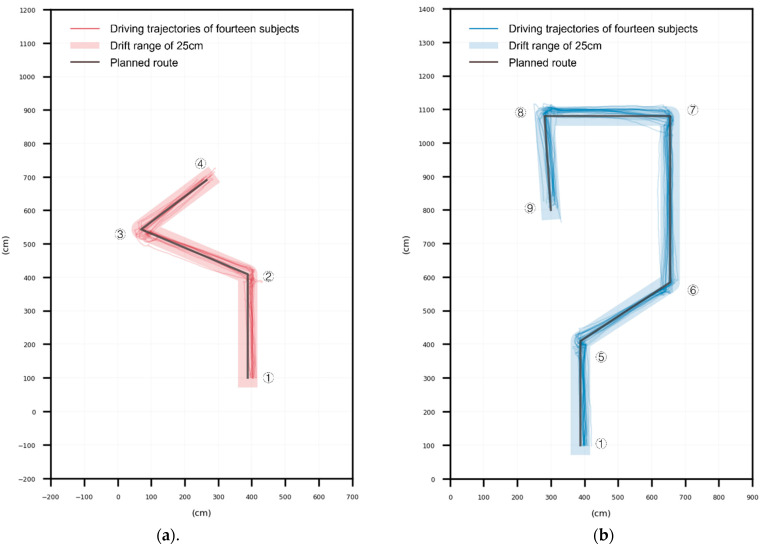
Driving trajectories of 14 subjects. (**a**) Short path driving trajectories of 14 subjects and (**b**) long path driving trajectory of 14 subjects.

Figure 11a displays the 14 subjects’ short path driving trajectories for a total of 14 times, while Figure 11b shows the long route driving trajectories of the 14 subjects for a total of 28 times. Figure 11 indicates that all subjects drove on the expected route and completed the driving evaluation. However, there was a drift between the intended route at point ① and the subject’s driving trajectory. This was because the driving test was carried out with the wheelchair’s center aligned with point ①, but the positioning label was fixed to the right rear of the wheelchair. Consequently, the trajectory at point ① appeared to be drifting to the right. Additionally, some trajectories still had large drifts, especially around the turning points, including locations ②, ③, ⑤, ⑦, and ⑧. Field observations and feedback from the subjects after the experiment revealed two main reasons for the turn deviations: (1) the laptop was improperly positioned, obstructing the subjects’ view and making it difficult for them to determine whether they had reached the target position, and (2) there was some deviation in the track record due to the jitter generated during the wheelchair driving process.

#### 4.2.2. User Experience Evaluation

Upon completing the experiment, the subjects were asked to fill out a SUS questionnaire based on their experience using the device, as well as a satisfaction questionnaire. The SUS consists of 10 questions, with each question rated on a scale of 1 to 5. To calculate the SUS score, the base value for each question, ranging from 0 to 4 points, was first determined. For positively worded questions with odd question numbers, the base value equaled the score for that question minus 1. For negatively worded questions with even question numbers, the base value equaled 5 minus the score for that question. Finally, the base values of the 10 questions were summed and multiplied by 2.5 to obtain the SUS score. According to a previous study [41], SUS scores can be interpreted as follows: a system with a score below 50 is unacceptable, a score of 50–70 is critical, and a score of 70 or higher is acceptable.

**Figure 12:**
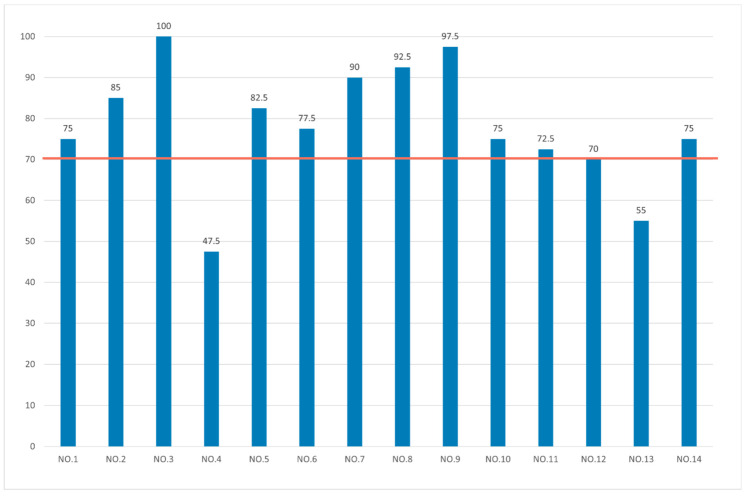
SUS scores for each subject.

The results indicated that the mean SUS score for the 14 subjects was 78.21. Based on the findings by Bangor et al. [41,42], a system is considered to have good usability when its SUS score exceeds 71.4. Figure 12 presents the SUS scores of all participants, with the orange line representing the threshold of 70 points. The graph clearly indicates that the majority of the 14 participants scored above 70, reflecting favorable usability perceptions. However, it is worth noting that two participants scored below 70, suggesting a lower level of perceived usability in their assessment. Through post-experimental interviews, we identified the following reasons for these lower scores:

1. The eye tracker occasionally failed to capture the gaze position after pressing the touch sensor (due to the subject blinking or gazing too far out of position), causing the system to report an error.
2. The eye tracker was positioned too close to the eyes, resulting in discomfort during extended use.
3. The voice and vibration feedback was not prominent enough and lacked a sense of security.

Ten of the fourteen subjects scored over 74.1 on the SUS, indicating that they found the system’s usability acceptable and reasonable. The subjects generally reported that the gaze-controlled wheelchair interaction was easy to understand and learn, and it effectively achieved the intended mobility improvements.

The satisfaction questionnaire comprised three questions, as illustrated in Table 11. The satisfaction questionnaire utilized a 5-point Likert scale. Based on the results in Table 11, the mean score for the three questions was 4.36, suggesting that the subjects were quite satisfied with the system and would be willing to recommend it to friends in need. The proposed gaze control wheelchair system fulfilled the subjects’ expectations.

Therefore, the future optimization direction and strategy for the gaze-controlled wheelchair are (1) ensuring system stability while shortening the delay time and enhancing the system’s timeliness, and (2) further optimizing the interaction mode to reduce the load on the user’s head and eyes, as well as improving the feedback system to increase feedback for different states, such as starting and turning.

#### 4.2.3. Discussion

The proposed gaze-controlled wheelchair system aims to support individuals with upper limb dysfunction who encounter difficulties utilizing traditional power wheelchairs with joysticks. Our system, founded on the extended FBS model and the MosCow method, facilitates natural sight interaction for wheelchair control, effectively eliminating the need for joysticks. Furthermore, the system addresses user safety and feedback concerns through the implementation of an emergency stop module, as well as voice and vibration modules. This design ensures responsiveness to user needs and concerns, promoting enhanced comfort and control. The efficacy of the proposed gaze-controlled wheelchair interaction system was demonstrated through an experiment involving 14 healthy participants. The collective results indicated a promising solution for disabled users. The participants expressed optimism regarding the system’s usefulness and ease of use, which allowed them to quickly learn and navigate to their desired destinations. Additionally, they proposed performance improvements for the gaze-controlled wheelchair, such as reducing the delay time and enhancing ride comfort.

The project is currently in its preliminary development stage. Healthy volunteers were selected to participate in the experiment to mitigate potential risks to disabled individuals due to the product’s immaturity. While this may impact the authenticity of the experimental results, it enables preliminary verification of the system. In the future, we plan to optimize the system’s functions and performance and involve users with limited upper limb mobility in the experiments to derive more accurate conclusions.

During the experiment, ensuring the accuracy and effectiveness of the eye-tracking device and program operation was of paramount importance. Consequently, a laptop was placed in front of the participants for device calibration and testing. Regrettably, the laptop’s presence interfered with users’ gaze behavior, necessitating that they extend their heads to view the path ahead. In future work, we aim to optimize and adjust our methodology to prevent such interference. To guarantee the accuracy and validity of the experimental results, we intend to explore alternative calibration methods or modify the laptop’s positioning. Furthermore, we will endeavor to minimize any potential sources of interference that may impact users’ gaze behavior. These improvements will facilitate more accurate and reliable experimental results, enabling a deeper understanding of the system’s capabilities and limitations.

## 5. Conclusions

This study presents a gaze-controlled system for power wheelchairs, utilizing the extended FBS model and the MosCow method. We began by conducting a comprehensive analysis of user requirements derived from literature research and user interviews, according to the extended FBS model. Subsequently, the MosCow method was used to prioritize and categorize the identified user requirements into “Must Have” and “Should Have” categories. Based on these requirements, we developed an intuitive and efficient gaze-controlled system for power wheelchairs, designed to assist individuals facing manual control challenges who struggle to use a joystick.

The proposed system aims to reduce complexity while addressing users’ essential needs. To achieve this, critical components such as eye trackers, control modules, audio and vibration modules, and touch sensors are integrated into existing power wheelchairs. The system utilizes a three-level approach of perception, decision making, and execution to allow for the continuous analysis of the user’s intention to move short distances, thus enabling long-distance driving. The system’s effectiveness was verified through gaze-controlled wheelchair driving experiments in a simulated indoor environment. All participants successfully followed the designated route, exhibiting an average drift of less than 20 cm. Moreover, the SUS results suggest that the system possesses good usability, while the satisfaction scale indicates that the subjects expressed high levels of satisfaction with the system.

However, our study has certain limitations. Firstly, due to the impact of COVID-19 and the product being in the initial development stage, we recruited healthy individuals with hands bounded as subjects. Secondly, the system was tested and evaluated exclusively indoors, limiting the assessment of its effectiveness and user experience in outdoor environments. Moreover, the study presents only a preliminary application of the extended FBS model without in-depth optimization and integration.

Despite these limitations, our research successfully demonstrates the process of transitioning from user research to a requirement analysis, design prototype implementation, and testing. By employing the extended FBS model and the MosCow method, we extracted and sorted users’ real requirements, culminating in a valuable concept prototype. We also conducted simulation tests to confirm the system’s effectiveness, usability, and user satisfaction. In future research, we plan to address these limitations and develop a wheelchair system that is more beneficial, easy to use, and accessible for individuals facing manual control challenges.

## Figures and Tables

**Table 1:** Overview of the aims and methods of the three phases of the current study.

Phase	Aim	Method
Phase 1	Defining the functional requirements of the system	Literature research, user interviews, extended FBS model
Phase 2	Realizing the functional requirements of the system	MosCow method
Phase 3	System evaluation	Simulation tests and system usability scale

**Table 2:** Summary of requirements.

Works	Methods	User Needs
[25]	AHP, Kano Model	Affordability, Comfort, Cost effectiveness, Optimal functionality, Innovation
[26]	Semi-structured interviews	Safety
[27]	Semi-structured interviews	Safety
[28]	Semi-structured interviews	Safety
[29]	Telephone questionnaire/interview	Comfort
[30]	Wizard-of-Oz approach	Safety, Easy to manipulate
[31]	Personal interviews	Safety, Comfort, Weight

**Table 3:** MosCow method.

Level	Name	Meaning
M	Must Have	Non-negotiable product needs that are mandatory for the team.
S	Should Have	Important initiatives that are not vital, but add significant value.
C	Could Have	Nice to have initiatives that will have a small impact if left out.
W	Won’t Have	Initiatives that are not a priority for this specific time frame.

**Table 4:** F^i^ and Re^i^ initially identified according to the MosCow method.

Level	F^i^	Level	Re^i^
S&C	Available wheelchair integration	S&C	High efficiency
S&C	Multimode feedback	S&C	Accurate control
S&C	Eye-control input	S&C	Intelligent assistance
M	Natural interaction	M	Conformity to custom
M	Emergency stop	M	Safety
S&C	Intelligent obstacle avoidance	S&C	Low cognitive loads
S&C	Cooperative control	M	Clear feedback
S&C	Path planning	S&C	Low cost

**Table 5:** Final requirement prioritization.

Level	F^i^	Level	Re^i^
M	Natural interaction	M	Conformity to custom
M	Emergency stop	M	Safety
S	Available wheelchair integration	M	Clear feedback
S	Multimode feedback	S	High efficiency
S	Eye-control input	S	Accurate control
C	Intelligent obstacle avoidance	S	Low cost
C	Cooperative control	C	Intelligent assistance
C	Path planning	C	Low cognitive loads

**Table 6:** Details of the sensors.

Name	Brand	Specification
Pupil Core headset	Pupil Labs	Pupil Core
Ultra-Wideband	WiT	JY1000-BU
Touch Sensor	KEYES	TTP223
Motor	XINHUITENGRC	MG995
Arduino module	Arduino	MEGA2560\UNO R3
Voice module	Risym	MH-PMD
Vibration module	Yahboom	YB-MVV07
Mobile Power	Shanke	SK1000A

**Table 7:** Driving times for short routes for 14 subjects.

Subject	Gender	Negative Diopters	Time (min)	Average Time (min)
NO.1	Male	4.5	1.31	1.13
NO.2	Female	4.6	2.19	
NO.3	Female	4.7	0.81	
NO.4	Male	4.7	1.11	
NO.5	Male	4.8	1.06	
NO.6	Male	4.6	0.75	
NO.7	Male	4.9	1.36	
NO.8	Male	5.0	0.96	
NO.9	Female	4.8	0.94	
NO.10	Female	4.4	0.96	
NO.11	Female	4.7	2.07	
NO.12	Female	4.6	0.84	
NO.13	Female	4.4	1.27	
NO.14	Female	4.9	0.90	

**Table 8:** Driving times for long routes for 14 subjects.

Subject	Gender	Negative Diopters	Time (min)	Average Time (min)
NO.1	Male	4.5	2.67	1.79
			1.95	
NO.2	Female	4.6	2.53	
			2.21	
NO.3	Female	4.7	1.54	
			1.73	
NO.4	Male	4.7	2.57	
			1.37	
NO.5	Male	4.8	1.17	
			1.43	
NO.6	Male	4.6	1.21	
			1.25	
NO.7	Male	4.9	1.36	
			2.05	
NO.8	Male	5.0	1.38	
			1.37	
NO.9	Female	4.8	1.31	
			1.34	
NO.10	Female	4.4 (Wears contact lenses)	3.52	
			1.36	
NO.11	Female	4.7	1.42	
			1.54	
NO.12	Female	4.4 (Wears contact lenses)	3.15	
			2.37	
NO.13	Female	4.6 (Wears contact lenses)	1.53	
			1.55	
NO.14	Female	4.8	1.48	
			2.39	

**Table 9:** The average drift of short path driving for 14 subjects.

Subject	Average Drift per Test (cm)	Average Drift (cm)
NO.1	16.77	19.49
NO.2	27.81	
NO.3	25.02	
NO.4	28.97	
NO.5	10.26	
NO.6	13.26	
NO.7	34.26	
NO.8	18.67	
NO.9	17.82	
NO.10	20.80	
NO.11	9.37	
NO.12	15.29	
NO.13	16.94	
NO.14	17.64	

**Table 10:** The average drift of long path driving for 14 subjects.

Subject	Average Drift per Test (cm)	Average Drift (cm)
NO.1	33.99	15.72
	11.82	
NO.2	18.88	
	13.98	
NO.3	14.50	
	10.85	
NO.4	19.48	
	19.93	
NO.5	13.87	
	13.26	
NO.6	16.39	
	19.7	
NO.7	16.49	
	13.87	
NO.8	16.50	
	18.96	
NO.9	12.98	
	17.72	
NO.10	9.79	
	12.39	
NO.11	10.64	
	12.16	
NO.12	32.89	
	7.98	
NO.13	12.08	
	12.40	
NO.14	12.67	
	14.12	

**Table 11:** Results of the satisfaction scale.

Question	Average Score	Max. Score	Min. Score
1. Overall, I am satisfied with the system	4.29	5	4
2. I would recommend it to a friend who needs it	4.43	5	3

## Data Availability

Available upon request.
